# Editors’ Book Table

**Published:** 1874-10

**Authors:** 


### Editors' Book Table.

[NOTE. — All works reviewed in the columns of the CHICAGO MEDICAL JOURNAL may be found in the extensive stock of W. B. KEEN, COOKE & CO., whose catalogue of Medical Books will be sent to any address upon request.]

*The Physiology of Man;* designed to represent the existing state of Physiological Science, as applied to the functions of the human body. By AUSTIN FLINT, Jr., M.D., Professor of Physiology and Physiological Anatomy in the Bellevue Hospital Medical College, N. Y., etc., etc. In five volumes. Vol. V. (with general index to the five volumes): Special Senses; Generation. New York: D. Appleton & Co. 1874.

It is needless to say more of this, the fifth and last, volume of Dr. Flint's work on Physiology, than that it comes fully up to its predecessors in all that has made them already the American physiologist's pride and boast. Upon the great physiological questions of the day the author will be found upon the right side, as illustrated by his remarks upon "so-called spontaneous generation," and elsewhere. We cannot follow him, however, with equal safety beyond these limits; for the occasional glimpses of his metaphysical notions with which we are favored, smack, too much of the all-pervading materialism of the day to warrant our endorsement. Taken as a whole, however, Flint's Physiology must be pre-eminently the text-book of the American student, with which he cannot dispense. H.

*A Treatise on Therapeutics,* Comprising Materia Medica and Toxicology, with especial reference to the Application of the Physiological Action of Drugs to Clinical Medicine. By H. C. Wood, Jr., M.D., Professor of Botany and Clinical Lecturer onDiseases of the Nervous System in the Medical Department of the University of Pennsylvania, etc., etc. Philadelphia: J. B. Lippencott & Co. 1874.

A step in the right direction, at last. A treatise on "Therapeutics;" not simply a catalogue of drugs and what they are good for; not simply a nosological table, and of drugs appropriate thereto; but an attempt to lay the foundation of a true science of therapeutics, with an admirable definition of the term, *i. e.*, "the application of the physiological action of drugs to clinical medicine." The author expresses in his preface a want which every other earnest student must echo, "the want of something more" than the standard treatises on materia medica and therapeutics, and asserts what we most heartily endorse, that "the old and tried method in therapeutics is that of empiricism, or, if the term sound harsh, of clinical experience;" and if his prefatory commentaries upon the status of therapeutics as a science be severe, they must nevertheless be admitted to be true.

His classification of remedial agents is simple and comprehensive, consisting of their division into systematic and non-systematic remedies, and the subdivision of the former into general and local remedies—which classification, like all others, he not inaptly terms "a convenient row of pegs upon which to hang our ideas and facts, so that they may be easily retained and easily accessible when wanted."

It is gratifying to perceive that the author confidently rests his therapeutical conclusion upon the data of experimental physiology and zoo-chemistry.

It would be a pleasant task, did space and time permit, to give here an extended and detailed analysis of the contents of this work. For while it assumes, occasionally, therapeutical propositions which can scarcely be considered as determined, and others, again, which we do not feel willing altogether to endorse, it, at the same time, elucidates others, hitherto indefinite, and supplies the data for the determination of still more. A careful study of Dr. Wood's book will leave upon the mind of the student the feeling of regret that it is not more detailed, that many important remedies have been omitted from consideration, the author having apparently been satisfied with the discussion of typical remedies. In our humble judgment, the book is altogether the best work on therapeutics in the language, and entitles its author to the sincere thanks of all who wish to escape the trammels of empiricism. H.

BOOKS RECEIVED.

*The Complete Hand-book of Obstetric Surgery.* By CHARLES CLAY, M.D. Philadelphia: Lindsay & Blakiston. 1874.

*A Manual of Surgical Emergencies.* By WILLIAM PAUL SWAIN, F.R.C.S. Philadelphia: Lindsay & Blakiston. 1874.

*Transactions of the Pathological Society of Philadelphia.* Vol. IX. 1871–72–73.

PAMPHLETS RECEIVED.

*Smithsonian Miscellaneous Collections.* No. 279. The Toner Lectures. Instituted to encourage the discovery of new truths for the advancement of medicine. Lecture III.: On Strain and Over-action of the Heart. By J. M. DA COSTA, M.D., Professor of Practice of Medicine in the Jefferson Medical College, Philadelphia; Physician to the Pennsylvania Hospital, etc. Delivered May 4, 1874.

*Transactions of the Linton District Medical Society of the State of Missouri.* First to fifth session, inclusive. Mexico, Mo., 1874.

*Transactions of the Michigan State Medical Society for the year* 1874. Series II. Vol. VI.

*The Hypodermic Use of Quinine a dangerous experimental Medication, and rarely justifiable.* By STEPHEN ROGERS. New York: D. Appleton & Co. 1874.

*The Temperance Question from the Standpoint of the Present.* Delivered by JNO. MORGAN MCKOWN, M.D. Semi-annual Address of the Æsculapian Society of the Wabash Valley. 1874.

*The Relations of Medical Societies to Progress in Medical Science.* Inaugural Address of the President of the Medical Society of the County of Kings, New York. ALEXANDER J. C. SKENE, M.D. June 16, 1874.

*Clinical Report of the Lying-in Service at Bellevue Hospital for the year* 1873. By WILLIAM T. LUSK, M.D., Professor of Obstetrics and Diseases of Children in Bellevue Hospital Medical College. Reprinted from the New York *Medical Journal*, Aug., 1874. D. Appleton & Co.

*Experimental Researches of the Physiological and Therapeutic Action of the Phosphate of Lime.* By L. DUSART, *Ex-interne des Hopitaux de Paris.* Paris: 113 Faubourg St. Honore. Price two shillings.

*Report of the Board of Health of the City of Philadelphia, to the Mayor, for the year* 1873. Philadelphia: Printed by order of the Board.

*Address of Joseph M. Toner, M.D., President of the Association.* Extracted from the Transactions of the American Medical Association. Philadelphia: Collins, Printer, 705 Jayne street. 1874.

JOURNALS RECEIVED.

The American Practitioner—August.
" Australasian Medical and Surgical Review—June.
" American Medical Weekly, Sept. 5, 12.
L'Anatomie et de la Physiologie, Journal de No. 5.
The Boston Journal of Chemistry—September.
" Canada Medical and Surgical Journal—August and September.
" Clinic, Cincinnati—August 15, Sept. 12.
" Cincinnati Lancet and Observer—Sept.
" Detroit Review of Medicine and Pharmacy—September.
" Druggists' Circular—September.
" Indiana Journal of Medicine—August and September.
" Kansas City Medical Journal—August.
" London Lancet—August.
" Medical Herald, Leavenworth, Kansas—Aug. 1, 15, Sept. 5, 12.
" Medical Examiner, Chicago—Aug. 15.
" Medical Times, Philadelphia—Aug. 15, 22, Sept. 5, 12.
" Medical and Surgical Reporter—Aug. 15, 22, 29.
" Medical Record, New York—Aug. 1, Sept. 1.

The Missouri Clinical Record—September.
" Medical Eclectic, New York—September.
" Medical Mirror, New York—September.
" New York Medical Journal—September.
" Nashville Journal of Medicine and Surgery—September.
" Progres Medical, Paris—Nos. 26, 27, 28, 30, 31, 32, 33, 34, 35.
" Pacific Medical and Surgical Journal—August and September.
" Psychological and Medico-Legal Journal—August.
" Practitioner, London—August.
" Pharmacist, Chicago—September.
" Physician and Pharmacist.
" Peninsular Journal of Medicine—September.
" Richmond and Louisville Medical and Surgical Journal—August.
' St. Louis Medical and Surgical Journal—September.
" Southern Medical Record—August.
" Texas Medical Journal, Galveston—July.
Transactions of the Medico-Chirurgical Society of the State of Maryland. Seventy-fifth Annual Session—April, 1874.
Transactions of the Medical Society of the State of West Virginia. Instituted April 10, 1867. Wheeling, 1874.
The United States Medical and Surgical Journal—July.
Vick's Floral Guide—No. 4, 1874.
The Virginia Medical Monthly—September.

